# Positive Clinical Signs in Functional Neurological Disorders: A Narrative Review and Development of a Clinical Decision Tool

**DOI:** 10.3390/brainsci15090997

**Published:** 2025-09-16

**Authors:** Ioannis Mavroudis, Katerina Franekova, Foivos Petridis, Alin Ciobica, Sotirios Papagiannopoulos, Dimitrios Kazis

**Affiliations:** 1 Department of Neuroscience, Leeds Teaching Hospitals, NHS Trust, Leeds LS13EX, UK; 2 Third Department of Neurology, Aristotle University of Thessaloniki, 54124 Thessaloniki, Greece; 3 Academy of Romanian Scientists, 3 Ilfov, 050044 Bucharest, Romania; 4 School of Medicine, University of Leeds, Leeds LS29JT, UK; 5 Department of Biology, Faculty of Biology, Alexandru Ioan Cuza University of Iasi, Carol I Avenue 20th A, 700505 Iasi, Romania; 6 Center of Biomedical Research, Romanian Academy, Iasi Branch, Teodor Codrescu 2, 700481 Iasi, Romania; 7 Preclinical Department, Apollonia University, Păcurari Street 11, 700511 Iasi, Romania

**Keywords:** Functional Neurological Disorder, Clinical Decision Tool, positive diagnostic signs

## Abstract

**Background**: Functional Neurological Disorders (FNDs) encompass a spectrum of disabling conditions, including functional limb weakness, tremor, gait disorders, seizures, and cognitive impairments. While previously diagnosed by exclusion, a growing consensus now supports the use of positive clinical signs as a basis for diagnosis. Despite this paradigm shift, frontline clinicians lack an integrated, accessible clinical tool for guiding diagnostic reasoning across FND subtypes. **Objectives**: This study aims to (1) synthesize the contemporary evidence on positive clinical signs across major FND subtypes and (2) develop a structured Clinical Decision Tool to support early and confident diagnosis in routine clinical settings. **Methods**: A focused narrative review was conducted using peer-reviewed publications and neurology reference texts, identifying reproducible positive clinical signs relevant to FND diagnosis. Signs were extracted, tabulated by subtype, and integrated into a modular decision-making framework designed for usability across outpatient, emergency, and specialist contexts. **Results**: The review identified 60+ positive signs across seven FND subtypes. These include Hoover’s sign for limb weakness, entrainment for tremor, variable responsiveness in NESs, and paradoxical memory performance in Functional Cognitive Disorder. A Clinical Decision Tool was developed, featuring subtype-specific checklists, diagnostic confidence indicators, and red flag alerts, and it is currently available in printable format. **Conclusions**: This study offers a novel, evidence-based decision tool to facilitate the positive diagnosis of FND. By consolidating observable signs into a practical format, it aims to reduce diagnostic delays, avoid unnecessary investigations, and enhance patient–clinician communication. Future efforts will focus on clinical validation and digital implementation.

## 1. Introduction

Functional Neurological Disorders (FNDs) represent a common, disabling, and historically misunderstood group of conditions characterized by neurological symptoms not explained by structural or degenerative pathology [1,2,3,4]. These disorders—ranging from functional weakness, tremor, and seizures to cognitive impairments—are increasingly recognized as having identifiable neurobiological, psychological, and behavioral underpinnings. Once relegated to diagnoses of exclusion, the field has advanced substantially through the identification of positive clinical signs that support a diagnosis based on observable inconsistencies or incongruities with known disease patterns [1,5,6,7,8].

Despite advances in understanding the neurobiology and phenomenology of FNDs, diagnostic delays remain prevalent, contributing to unnecessary investigations, prolonged disability, and patient distress [9,10]. This is especially relevant in busy clinical settings such as neurology clinics and emergency departments, where physicians may lack training in recognizing the nuanced signs that distinguish functional from organic disease. Up to 16% of new neurology referrals and over 40% of patients in specialist memory clinics present with symptoms ultimately diagnosed as functional in origin [2,11].

A key shift in modern FND diagnosis has been the emphasis on positive signs—such as Hoover’s sign in functional weakness, entrainment in functional tremor, and asynchronous limb movements in functional nonepileptic seizures (NESs)—that offer internal inconsistency or incongruity as diagnostic evidence [12,13]. Similarly, in Functional Cognitive Disorder (FCD), findings such as better delayed than immediate recall, normal test scores despite severe complaints, and signs like attending clinic alone or bringing a written list of symptoms provide strong support for diagnosis [9,11].

However, while the diagnostic literature is expanding, there remains a lack of structured clinical tools that compile these signs into a usable decision-making framework for frontline clinicians. Most current frameworks focus on subtype-specific reviews or single-symptom analysis. To our knowledge, no comprehensive clinical decision aid currently exists that integrates the positive diagnostic features of multiple FND subtypes into a unified, accessible format.

## 2. Aims of This Study

This review addresses that gap by

1. Synthesizing evidence from contemporary literature on positive clinical signs across FND subtypes, including motor, cognitive, sensory, gait, and seizure presentations.
2. Developing and presenting a structured Clinical Decision Tool that incorporates subtype-specific diagnostic signs, intended for use in outpatient, emergency, and specialist clinical contexts.
3. Proposing a scalable, modular format for this tool—beginning with a printable checklist and extending to potential integration in digital platforms.

Through this dual focus—systematic review and tool development—we aim to contribute to earlier, more confident diagnoses, promote appropriate referrals, and support evidence-based reassurance and communication with patients. The overarching goal is to shift FND care from uncertainty and exclusion to positive identification, structured decision-making, and timely intervention.

## 3. Methodology

### 3.1. Study Design

This study employed a two-pronged approach comprising

1. Narrative Review of clinical literature on diagnostic features of Functional Neurological Disorders (FNDs), with emphasis on positive signs and internally inconsistent clinical findings.
2. Development of a Clinical Decision Tool, structured from evidence synthesized across multiple FND subtypes, designed for practical implementation by neurologists, psychiatrists, general practitioners, and emergency physicians.

### 3.2. Literature Search Strategy

We conducted a focused narrative review of peer-reviewed publications and major neurology reference texts with an emphasis on identifying reproducible positive clinical signs in the diagnosis of FND. Peer-reviewed publications retrieved from PubMed using structured queries (e.g., “functional neurological disorder positive signs”, “diagnostic signs of functional tremor”, “clinical features of functional cognitive disorder”) without filtering by study type.

### 3.3. Inclusion and Extraction Criteria

Included studies focused on diagnostic features of FND, including Functional Movement Disorders (FMDs), Functional Seizures (NESs), Functional Cognitive Disorder (FCD), Functional Sensory Symptoms, Functional Gait, and Functional Dystonia.

In addition, the selected studies explicitly described or tested for positive clinical signs commonly used in diagnosing FND, such as Hoover’s sign, distractibility, entrainment, and discrepancies between clinical tests and patient-reported symptoms.

To ensure relevance and quality, we only included studies that provided original data, consensus-based expert opinion, or comprehensive reviews related to these diagnostic signs.

From each source, the extracted information included the clinical sign or feature described, the specific FND subtype it applied to, and any reported diagnostic performance metrics such as sensitivity, specificity, or likelihood ratios.

### 3.4. Synthesis and Table Development

Extracted findings were synthesized into structured tables by subtype, capturing the sign name, clinical description, diagnostic implication, and citation.

This tabulated synthesis formed the basis for the clinical decision aid prototype, allowing each FND subtype to be represented with its associated diagnostic signs.

### 3.5. Tool Development

Using the evidence-based tables, we designed a Clinical Decision Aid intended for use in clinical settings. Key steps included

1. Clustering signs under diagnostic categories (e.g., functional limb weakness, tremor, FCD).
2. Organizing findings into a modular checklist with concise phrasing.
3. Converting the content into an accessible PDF reference guide.
4. Structuring the tool to allow future translation into app-based or electronic decision support formats.

Formatting and design were guided by principles of clinical usability—i.e., simplicity, clarity, and reliance on observable signs.

### 3.6. Ethical Considerations

This study did not involve direct patient data and did not require institutional ethical approval. All sources used were publicly available, peer-reviewed publications or published textbooks.

## 4. Results

The narrative review identified a literature base comprising both original research studies and review articles, predominantly narrative and expert reviews. This body of evidence yielded 54 positive clinical signs and diagnostic features across seven primary subtypes of Functional Neurological Disorder (FND). The findings are organized below by subtype and summarize both clinical signs and diagnostic indicators with supporting references.

### 4.1. Functional Limb Weakness

Functional limb weakness can be reliably identified through a range of positive clinical signs that reveal inconsistencies with neuroanatomical principles and expose the preserved capacity for voluntary motor function despite reported disability. These signs help differentiate functional weakness from organic paresis and provide a foundation for a confident diagnosis.

One of the most widely used bedside tests is Hoover’s sign, which assesses hip extension strength in the supine position. When a patient is asked to flex the contralateral hip against resistance, involuntary extension of the allegedly weak limb is often observed. This phenomenon demonstrates preserved motor function inconsistent with true paresis and is considered a hallmark sign of functional weakness [2,14].

A related but less commonly used maneuver is the Abductor sign, which functions on similar principles. When the patient attempts to abduct the contralateral hip, the supposedly weak hip shows unexpected activation or strength, again suggesting non-organic pathology [14].

Another key finding is collapsing weakness (also known as “give-way weakness”). Here, the patient initially offers resistance to the examiner’s pressure but then suddenly allows the limb to collapse, without following the expected mechanical behavior seen in true neurological deficits. This sign lacks the organic pattern of resistance and indicates inconsistency under repeated testing conditions [1,15].

Entrainable weakness is another valuable sign. In this case, the patient’s strength or muscle tone changes when they are asked to perform a different rhythmic movement with another limb (e.g., tapping), leading to entrainment or coordination between the two tasks [6]. This response is atypical of organic weakness and can be particularly revealing.

Similarly, give-way weakness or inconsistent effort is observed when a patient displays fluctuating strength during repeated attempts or shows improved performance when distracted [2]. These findings reinforce the diagnosis when strength appears impaired only under conscious attention but normalizes when the patient is unaware they are being tested.

Finally, the presence of normal movement during emergencies or under distraction—such as using the limb to catch a falling object or stabilizing themselves during a loss of balance—can serve as compelling evidence. These preserved automatic responses contradict the experienced deficit and further support functional etiology [6].

Together, these signs form a robust framework for the positive identification of functional limb weakness. Their value lies not only in their diagnostic utility but in their capacity to shift the clinical approach away from exclusionary reasoning toward a model grounded in reproducible, observable phenomena [2,14].

### 4.2. Functional Tremor

Functional tremor is the most prevalent subtype of functional movement disorders and is distinguished by a range of positive clinical signs that highlight its inconsistency, variability, and sensitivity to external modulation. These signs contrast sharply with the regular, predictable patterns seen in organic tremor syndromes such as Parkinsonian or essential tremor, and their identification is key to forming a positive, confident diagnosis.

A core diagnostic maneuver is the entrainment test, which assesses the interaction between the tremor and voluntary movement of the contralateral limb. In this test, the patient is asked to mimic a rhythmic tapping movement with the limb opposite the one experiencing tremor. In functional tremor, the frequency of the tremor often shifts to match the voluntary movement, or the tremor may even become temporarily suppressed [5,16]. This entrainment reflects the functional underpinnings of the movement and strongly supports a functional diagnosis.

Closely related is the phenomenon of tremor inhibition or pause, where voluntary movements on the unaffected side—such as a tapping or clapping task—lead to a temporary reduction in or cessation of the tremor. This observation is incompatible with pathophysiological tremors, which typically persist regardless of contralateral movement [7,16].

Distractibility is another hallmark feature. Patients with functional tremor often exhibit reduced tremor amplitude or complete cessation while engaged in cognitively demanding tasks or when their attention is diverted. For instance, counting backward or engaging in a conversation may cause the tremor to fade. This variability under distraction is a strong positive indicator of functional etiology and offers a simple yet powerful bedside tool for diagnosis [7].

The co-activation sign offers an electrophysiological clue, characterized by simultaneous contraction of both agonist and antagonist muscle groups before tremor onset. This contrasts with the reciprocal activation typically observed in organic tremors and suggests an abnormal pattern of central motor control [5].

Additionally, variable frequency and axis—where the tremor frequency fluctuates or the axis of movement shifts between assessments—further distinguish functional tremor from the fixed rhythm and consistent axis seen in neurodegenerative tremor disorders [16,17].

Taken together, these signs—entrainment, distractibility, tremor inhibition, coactivation, and variability—comprise a constellation of features that are not only clinically observable but also highly specific. Their presence provides the foundation for a positive diagnosis of functional tremor and helps avoid unnecessary investigations or misdiagnosis [5,7,16].

### 4.3. Functional Jerks and Paroxysmal Movement Disorders

Functional myoclonus is a subtype of functional movement disorders characterized by jerky, non-rhythmic movements that differ fundamentally from organic myoclonus in both phenomenology and underlying physiology. Through careful clinical and electrophysiological assessment, several positive signs can help distinguish functional myoclonus from its organic counterparts.

One of the hallmark observations is the variable onset and pattern of jerks. In contrast to the stereotyped, fixed anatomical distribution of organic myoclonus, functional jerks tend to vary across time in their location, intensity, and frequency [18]. A patient may exhibit rapid movements in one limb during one session and a different limb or body part in another. This variability is not typical of myoclonus resulting from neurodegenerative or epileptic etiologies.

Precipitating factors further differentiate functional myoclonus [19]. Episodes are often triggered by psychological stimuli such as startle, heightened emotional states, or attentional focus on the affected body part. Such hyperattention or internal anticipation can intensify the movements, lending further support to a functional diagnosis. The link between psychological stress and symptom provocation underscores the need for a biopsychosocial framework in assessment.

Another important feature is the absence of reflex correlates. Organic myoclonus is frequently tied to physiological reflex circuits—such as cortical, subcortical, spinal, or brainstem loops—and typically follows consistent timing and reflex latency patterns. Functional jerks, by contrast, lack such correlates [18]. Electrophysiological testing, including surface EMG and EEG, often fails to reveal any abnormal reflex transmission or latency.

Electrophysiological studies have also examined the presence of pre-movement potentials, especially the Bereitschaftspotential or contingent negative variation—a negative slow cortical potential that precedes voluntary movement. In functional myoclonus, such potentials may be present, suggesting a degree of volition or psychophysiological preparation. However, they are often inconsistent or variably expressed, making them suggestive rather than definitive [20].

Collectively, these findings—variability in onset and pattern, presence of psychological precipitants, lack of physiological reflex circuitry, and inconsistent pre-movement cortical potentials—help to define functional myoclonus as a distinct clinical and electrophysiological entity. Recognizing these features enables clinicians to make a positive diagnosis and initiate appropriate multidisciplinary interventions, rather than pursue unnecessary investigations or misdiagnose epilepsy or neurodegeneration.

### 4.4. Functional Parkinsonism

Functional Parkinsonism (PP) mimics idiopathic Parkinson’s disease (PD) but is distinguished by a cluster of positive signs reflecting internal inconsistency, incongruity with organic patterns, and responsiveness to distraction or suggestion. Though rare compared to other functional movement disorders, it is critical to identify PP early to prevent unnecessary treatment escalation, including dopaminergic therapy or invasive interventions.

A defining feature of PP is the abrupt and maximal onset of symptoms, which sharply contrasts with the gradual, insidious progression of idiopathic PD. Patients frequently report severe disability from the outset, such as sudden-onset tremor or gait impairment, most commonly affecting the dominant hand [21,22,23].

Tremor in PP exhibits hallmark discrepancies from that of PD. It is typically present across all postures—rest, posture, and action—rather than being confined primarily to rest. It lacks the re-emergent component of PD tremor that pauses briefly with outstretched posture before resuming [24]. Instead, the tremor often intensifies when it becomes the focus of attention or when patients are queried about it. Distractibility is a key sign: tremor amplitude frequently diminishes or resolves during contralateral limb movement, mental arithmetic, or tandem walking. In some cases, entrainment is observed, where the tremor synchronizes with voluntary rhythmic movements of the opposite limb [7,16].

Variability is another diagnostic cornerstone. Tremor in PP may change in amplitude, frequency, and axis, even within the same examination—shifting from pronation/supination to flexion/extension patterns. Notably, finger tremor is often absent, helping distinguish PP from PD and essential tremor [25,26].

Muscle tone examination in PP reveals active resistance without true rigidity, particularly lacking the cogwheeling typical of PD. Resistance often diminishes with distraction, which is again the opposite pattern of organic Parkinsonism. Similarly, bradykinesia—slowness in movement—may be reported or demonstrated, but lacks the decrement in amplitude and velocity across repeated rapid movements that defines PD bradykinesia [22]. Patients may exhibit excessive effort, grimacing, or sighing during simple tasks, further pointing toward a functional etiology.

Gait findings in PP often include a slow, stiff walk, but without classic freezing of gait. There may be disproportionate responses during postural testing, such as flailing of arms or dramatic reeling in response to mild perturbation, without actual falls. Functional features like astasia–abasia, buckling knees, or a bouncy gait may co-occur, underscoring the overlap with other psychogenic gait presentations.

Speech may also be altered, ranging from stuttering and whispering to gibberish or “baby talk,” none of which aligns with neurodegenerative Parkinsonian syndromes. Notably, handwriting in PP is slow but lacks micrographia, the hallmark decrement in size over time that typifies PD [27].

### 4.5. Functional Gait Disorder

Functional gait disorders (FGDs) are among the most visibly striking manifestations of Functional Neurological Disorders (FNDs). They present with a wide range of abnormal walking patterns that often appear bizarre or exaggerated, yet they are produced without identifiable structural or neurological lesions. The diagnostic process for FGD relies not on exclusion but on the recognition of positive signs, inconsistencies, and incongruities—both within the gait pattern itself and between the patient’s reported symptoms and observed abilities.

One of the hallmark features of functional gait disorders is their sudden onset, often occurring in the absence of precipitating trauma or neurological injury. Generally, patients with FGD describe an abrupt onset of their walking difficulty [28]. The progression may be unusually rapid, sometimes escalating to severe disability within hours or days—a pattern atypical of organic neurologic gait disorders, which generally evolve more gradually.

A defining diagnostic clue is the inconsistency of gait performance, either within the same examination or across time. Patients may exhibit dramatic differences in walking ability in different contexts—for example, walking from the parking lot without aid but requiring a wheelchair in the clinic. This intraindividual variability is virtually never seen in purely neurological gait disorders [29].

The phenomenological spectrum of FGD is broad. Patients may mimic a variety of known gait abnormalities, but with atypical or exaggerated features. Observed gaits may include (1) hemiparetic or paraparetic gaits without consistent pyramidal signs or (2) a “walking on ice” pattern, which appears as an overly cautious gait with extremely short steps and a wide base, often accompanied by exaggerated balance reactions. Another gait type in FGD may be Truncal myoclonus or astasia–abasia, where patients sway dramatically or flail the limbs but manage to avoid falls, indicating preserved protective reflexes. Legs may also remain rigid, but postural reflexes and turns are performed effectively in “Robot-like” stiff-legged walking. Leg dragging with preserved strength on direct testing may also be observed, along with collapsing weakness when a leg gives way without an organic pattern.

These gait types often lack congruence with any known lesion pattern and demonstrate internal contradictions upon repeat testing or distraction [29,30].

Several positive clinical signs have been proposed and studied for their diagnostic utility in FGD:

The “Huffing and Puffing” Sign is a well-known sign where patients may groan, hyperventilate, or visibly strain while walking. Laub et al. (2015) found this sign present in 44% of patients with functional gait disorders and noted its high specificity (89–100%) when pain was not the primary symptom [31].

In the Chair Test, patients who appear unable to walk independently may still demonstrate good leg coordination when asked to propel themselves on a wheeled office chair [23,30,32]. This test also helps differentiate FGD from Parkinsonian gait [33].

A paradoxical reduction in sway or improved balance during mental distraction tasks (e.g., backward number tracing on the patient’s back) has been observed and quantitatively confirmed in studies using postural sway analysis [34,35] and is known as the Improvement with Distraction sign.

Lastly, patients with FGD demonstrate incongruent responses to novel gait tasks: patients may show exaggerated slowness or hesitancy when asked to perform specific tasks like walking backward or tandem walking, with overreactions that suggest voluntary modulation [29].

Functional gait disturbances may coexist with other functional neurological signs, including functional limb weakness, functional tremor, or sensory symptoms. The combination of unexplained motor and sensory features—especially if fluctuating or inconsistent—further supports a functional diagnosis [36].

### 4.6. Functional Visual Loss (FVL): Clinical Features and Positive Findings

Functional visual loss (FVL), or non-organic visual loss, encompasses visual complaints—such as decreased acuity, field constriction, or complete blindness—that are inconsistent with known anatomical or physiological patterns and unsupported by objective clinical or imaging findings. These symptoms may emerge in isolation or as part of a broader Functional Neurological Disorder (FND) and are recognized through positive signs rather than merely the absence of organic disease.

Patients with FVL often report sudden or dramatic visual symptoms, such as bilateral blindness or disabling field deficits, that lack correlation with ocular or neuro-ophthalmologic pathology. Notably, there is frequently a history of acute psychosocial stress, trauma, or comorbid FND symptoms—though patients may not explicitly link these factors [36].

A hallmark of FVL is the presence of visual ability during spontaneous behavior, despite profound reported deficits. For example, individuals claiming total blindness may navigate around obstacles, avoid collisions, or reach for objects with accuracy. These inconsistencies often emerge when the patient is unaware of being observed, supporting the functional etiology [36].

Several positive clinical signs aid in positively diagnosing FVL:

Tunnel Vision with Fixed Field Diameter is one of the positive signs indicating a FVD diagnosis. Patients may show visual fields of the same diameter at both near and far distances—an anatomically impossible finding that distinguishes FVL from true optic neuropathies or retinal disease [36].

Secondly, the presence of Optokinetic Nystagmus (OKN) in patients experiencing blindness indicates functional integrity of the visual pathways and cortical processing [31].

The Mirror Sign is another indicator for FVD. Patients may use mirrors appropriately for grooming or makeup application, suggesting intact functional vision despite complaints of severe visual loss [37].

Preserved Reflexes and Normal Visual Evoked Potentials (VEPs) in patients experiencing visual disturbance further differentiate FVD from optic nerve or chiasmal pathology [31,34].

Lastly, the ability to navigate obstacles points towards a functional cause when overt blindness is reported [38,39].

The diagnostic strategy of FVL, as with other functional disorders, rests on identifying internal inconsistencies and positive findings. FVL is not a diagnosis of exclusion, but rather a syndrome identifiable through structured observation and clinical reasoning. Assessments such as the “walking while blind” test and visual tracking during distraction can all reveal subtle indicators of intact vision [30,38].

### 4.7. Functional Cognitive Disorder (FCD)

Functional Cognitive Disorder (FCD) presents a unique profile of cognitive complaints that diverge from the patterns typically seen in neurodegenerative disorders. The diagnosis is not simply one of exclusion, but can be established through the identification of positive signs—particularly inconsistencies between reported symptoms, objective performance, and behavior in clinical settings [6].

A hallmark feature of FCD is better performance on delayed recall tasks compared to immediate recall, a reversal of the typical pattern seen in neurodegenerative diseases such as Alzheimer’s disease, where early impairment in consolidation affects delayed recall most severely. This paradoxical performance profile can be highly indicative of a functional basis for the reported memory complaints [40].

Another notable diagnostic clue is the presence of normal cognitive screening scores, such as on the MoCA (Montreal Cognitive Assessment) or ACE-R (Addenbrooke’s Cognitive Examination–Revised), despite the patient’s insistence on experiencing significant cognitive deficits. In the study by Ball et al. (2020), such discrepancies were characteristic of individuals with FCD, reflecting a disconnect between subjective experience and objective cognitive functioning [9].

Several behavioral and contextual markers further support the diagnosis. For instance, patients with FCD are significantly more likely to attend memory clinics unaccompanied, with an odds ratio of 8.7 for being diagnosed with FCD compared to other cognitive disorders [11]. This finding contrasts with patients with neurodegenerative conditions, who more commonly require support or accompaniment due to genuine cognitive and functional decline.

A particularly highly specific sign is the presentation of a written list of cognitive complaints. This behavior, while rare among patients with neurodegenerative disease, appears to reflect a meticulous metacognitive concern about memory performance. This sign has a specificity of 98% for FCD, underscoring its diagnostic utility [9].

Additionally, negative self-appraisal of memory despite preserved objective performance—sometimes referred to as “memory perfectionism”—is frequently observed. Patients with this trait exhibit heightened concern about any perceived lapses, even when these fall within normal ranges of cognitive function [41]. This contrasts with individuals suffering from true cognitive decline, who may demonstrate anosognosia, or lack of awareness of their deficits.

Another important sign is the absence of “head-turning” behavior toward a companion for help or corroboration during clinical interviews, a behavior often seen in patients with genuine cognitive impairment and awareness deficits [42]. The lack of such behavior in FCD suggests that the patient’s cognitive self-monitoring remains intact.

Collectively, these features illustrate that the diagnosis of FCD rests not merely on the absence of organic pathology, but rather on the positive identification of cognitive behavioral inconsistencies and patterns of heightened metacognitive concern. As emphasized by both Ball et al. (2020) and Cabreira et al. (2023), this growing body of evidence supports a structured and empirically informed diagnostic approach to FCD, moving beyond traditional exclusion criteria [9,11].

### 4.8. Functional Nonepileptic Seizures (NESs)

Nonepileptic Seizures (NESs), previously known as psychogenic seizures, are paroxysmal events that mimic epileptic seizures but lack the characteristic electrographic discharges on EEG. Rather than being diagnosed purely by exclusion, NESs are now recognized as a condition that can be positively identified through distinct clinical signs and behaviors observed during the events. These features, especially when combined with video–EEG confirmation, form a robust diagnostic framework [43,44].

A hallmark of NESs is the presence of asynchronous bilateral limb movements, particularly thrashing or flailing that is out of phase between the limbs. This contrasts with the typically rhythmic and synchronized motor activity of generalized tonic–clonic seizures. Another key sign is side-to-side head movement, often rhythmic and prolonged. This motion is rare in true epileptic seizures and has been considered a distinguishing feature [43]. These motor patterns, although dramatic, tend to lack the stereotyped consistency seen in epilepsy.

Prolonged duration of events is also characteristic of NESs. While epileptic seizures—especially tonic–clonic seizures—rarely last beyond one or two minutes, NES episodes frequently extend much longer, often with variable motor behaviors throughout [15,43]. Additionally, motor phenomena such as pelvic thrusting or rolling movements are more commonly seen in NESs and are typically absent in genuine epileptic events [43].

Ocular findings further aid in the diagnosis. In contrast to epileptic seizures, where eyes are usually open during the ictal phase, patients with NESs often keep their eyes closed throughout the event [45]. Resistance to passive eye opening by the examiner is another important indicator—such active opposition suggests retained voluntary control and awareness during the episode [46].

Patients may also demonstrate variable responsiveness during an episode, inconsistently reacting to verbal commands or tactile stimuli [44]. This contrasts with the impaired consciousness of generalized seizures, where the patient is typically unresponsive. Moreover, lack of postictal confusion, such as being alert immediately following the episode or recalling details about the seizure, suggests a nonepileptic nature.

Additional diagnostic signs include the absence of secondary features often seen in generalized tonic–clonic seizures, such as tongue biting, urinary incontinence, and injury from falls [45]. Their absence—especially in recurrent and dramatic seizures—should prompt consideration of NESs.

## 5. Clinical Decision Tool for Functional Neurological Disorders (FNDs)

### 5.1. Purpose and Design

The Clinical Decision Tool developed in this study provides a structured, evidence-based framework to assist clinicians in the positive diagnosis of Functional Neurological Disorders (FNDs) across multiple subtypes. It was designed to bridge the gap between research and real-world practice by consolidating reproducible positive clinical signs into a practical, modular format usable at the bedside, in outpatient clinics, and in emergency or acute care settings.

The tool builds upon the accumulating body of literature supporting the use of incongruent, inconsistent, and non-anatomical signs in the diagnosis of FND, a shift that allows clinicians to move beyond exclusion-based diagnoses.

### 5.2. Structure of the Tool

The decision tool is stratified by FND subtype and includes the following components:

**Subtype-Specific Panels:** Each subtype—Functional Weakness, Tremor, Jerks, Parkinsonism, Gait, Sensory Symptoms, Seizures (NESs), and Cognitive Disorder—is represented in a separate section with key diagnostic signs.

**Positive Sign Checklist:** For each condition, observable positive signs are listed along with brief operational definitions and references.

**Diagnostic Confidence Indicators**: Signs are tagged with their known specificity or utility where available (e.g., high-specificity signs such as Hoover’s sign, written complaint lists in FCD).

**Use Case Guidance:** The tool includes interpretive notes suggesting when signs are most useful (e.g., at first consultation, during video–EEG, or after normal imaging).

**Red Flag Box:** Each subtype includes warning signs that may indicate organic disease and necessitate further investigation.

The tool is currently available in a printable checklist format and is intended to support the initial diagnostic formulation, specialist referral justification, patient explanation and education, and the audit and training frameworks in clinical teams.

### 5.3. Diagnostic Flow

The tool supports a decision-making process based on the principle of positive pattern recognition:

**1.**  **Assess reported symptom;**
**2.**  **Match to relevant FND subtype;**
**3.**  **Observe for reproducible positive signs;**
**4.**  **Evaluate consistency, specificity, and distribution;**
**5.**  **Support diagnosis if ≥1 high-yield positive sign is present AND no red flag signs are identified.**

The presence of multiple convergent signs (e.g., entrainment + distractibility + coactivation in tremor) increases diagnostic confidence.

The current version serves as the foundation for these advancements and is intended to enhance clinician confidence, reduce unnecessary investigations, and improve early recognition of FND.

Table 1 includes an exhaustive list of the clinically observable positive signs of FND extracted from the literature reviewed in this paper. It does so in a concise manner as signs are organized by subtype, supporting a positive diagnosis of Functional Neurological Disorder (FND). Each sign reflects internal inconsistency or incongruity with structural disease and is linked to published sources.

**Figure 1:**
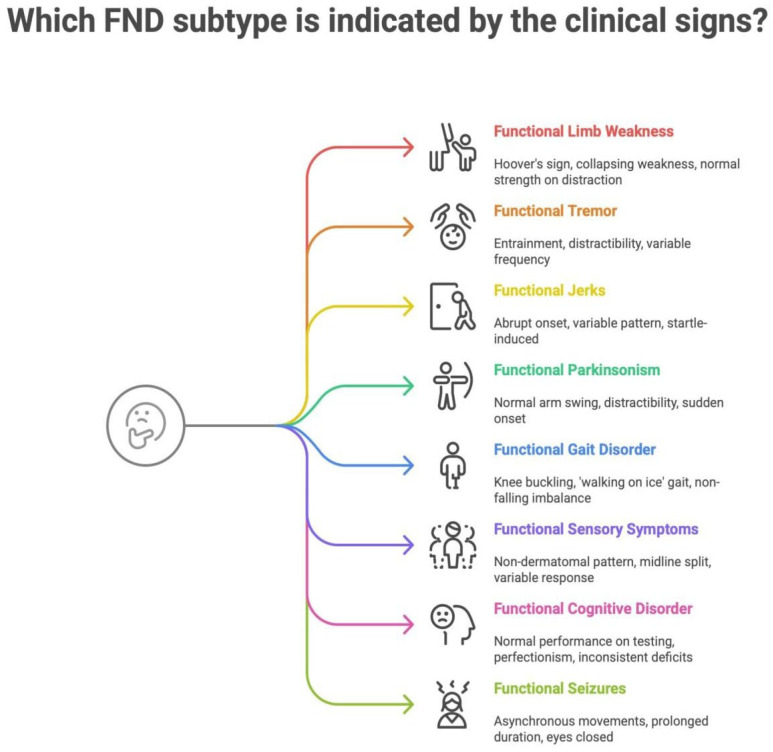
Mapping clinical signs to FND subtypes.

The following infographic (Figure 1) further illustrates the major Functional Neurological Disorder (FND) subtypes and their most common positive clinical signs. It aims to integrate both readability and ease of understanding into a summary tool. Each sign highlights characteristic inconsistencies or incongruities that support a functional diagnosis.

**Figure 2:**
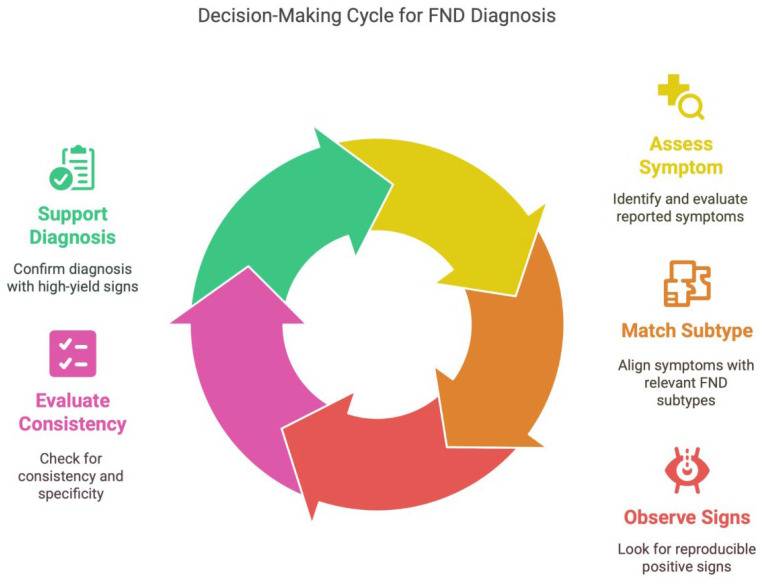
Decision-making cycle for FND diagnosis.

A clinical workflow for diagnosing FND is presented in Figure 2, emphasizing a structured approach: assess symptoms, match them to FND subtypes, observe for positive signs, evaluate their consistency, and use them to support a positive diagnosis. This figure is designed to guide clinician decision-making in the diagnostic process, along with Table 1 and Figure 1, which specify the FND types and positive signs to look for.

## 6. Discussion

The accurate and early diagnosis of Functional Neurological Disorders (FNDs) remains a clinical challenge, despite growing awareness and improved understanding of their neurobiological underpinnings. Historically, FND has been diagnosed via exclusion, relying heavily on negative tests and the absence of structural abnormalities. However, recent consensus [2,3] and diagnostic frameworks increasingly emphasize positive clinical signs, which are findings that are internally inconsistent, anatomically incongruent, or reversible under distraction.

While the presence of a positive clinical sign greatly strengthens the diagnostic confidence for Functional Neurological Disorder (FND), it is important to note that no single sign is pathognomonic. Many signs, such as give-way weakness, excessive effort, or gait inconsistency, may also be observed in other neurological or non-neurological conditions, including chronic pain, musculoskeletal disorders, or even malingering. For instance, collapsing weakness might also appear in patients with pain-related inhibition of movement or joint instability, without indicating a functional etiology. Similarly, variability in tremor or jerky movements can sometimes be seen in patients with severe anxiety or medication side effects. Therefore, while positive signs provide powerful diagnostic anchors, their interpretation must always be contextualized within the broader clinical picture, including patient history, symptom evolution, and collateral findings. This reinforces the value of clinical judgment and a biopsychosocial framework rather than reliance on single signs in isolation.

Our review consolidates more than 60 such signs across major FND subtypes, enabling a positively defined diagnostic approach. Importantly, the signs included in this tool are observable at the bedside, do not require specialized equipment, and have been supported by literature in neurology, neuropsychiatry, emergency medicine, and cognitive disorders.

Several recent publications have advanced guidance for FND with an emphasis on positive clinical signs. For instance, Perez et al. (2021) published a review and expert opinion on the assessment of motor FND specifically [18]. The narrative review by Finkelstein et al. (2021) proposes the positive signs and common diagnostic pitfalls along with an approximate reliability rating; however, it is not structured into a clinical checklist [10]. Another example would be the FCD diagnostic checklist by Cabreira et al. (2025) [47], who utilized multiple study methods to arrive at a formal tool differentiating FCD from other neurocognitive disorders. By contrast, our review was designed to consolidate positive signs across all major FND subtypes, extracting them from the wider literature and incorporating clinical reliability where available. Rather than focusing on a single presentation, our tool integrates motor, sensory, gait, and cognitive domains into a unified framework. This breadth enhances its utility in general neurology and emergency settings, where patients often present with mixed or evolving symptoms.

The inclusion of Functional Cognitive Disorder (FCD) represents a major advancement. Often missed or misdiagnosed as early dementia, FCD is now recognized as a distinct subtype of FND [9,11], with signs such as normal test performance despite severe complaints, a perfectionist cognitive profile, and behavioral cues like attending appointments alone or bringing detailed symptom lists.

Our tool’s design also addresses gaps in current practice by providing clinician-facing criteria that avoid ambiguous psychological language, as well as offering evidence-backed guidance to reduce misdiagnosis and over-investigation. Furthermore, this tool may be particularly useful in supporting clinical reasoning in uncertain settings like emergency departments and general neurology clinics.

Notably, this tool could help patients better understand their diagnosis when framed around consistent, reproducible signs rather than vague or psychosomatic explanations.

Future research should focus on validating these positive clinical signs as well as the proposed Clinical Decision Tool through quantitative scoring. This would strengthen the reliability of the tool in clinical practice and provide more direction regarding which signs to test for initially, depending on the patient’s symptom profile. Furthermore, future studies could also include investigations such as the electrophysiological aids [48] to complement the tool, although these require specialized equipment that may not be available in first-contact settings. Finally, integration of this tool into training curricula and digital platforms may further enhance clinician confidence and standardization in FND diagnosis, improving diagnostic delays and patient outcomes.

## 7. Limitations

Several limitations should be acknowledged.

Firstly, this paper was conducted as a narrative rather than a systematic review; therefore, some signs or publications may not have been captured. Future work could incorporate systematic methods and meta-analysis to estimate pooled diagnostic accuracy. Additionally, narrative reviews may also facilitate potential bias in the selection and synthesis of the literature, and as only English-language sources were included, relevant studies may have been excluded.

A second limitation is the variability in evidence strength. Not all signs have been validated in large prospective cohorts. Many derive from case series or expert opinion [14,24,25], while other signs were supported by original research [7,13,16,34,35]. Some foundational studies are dated and limited by the absence of standardized diagnostic criteria at the time [13,27,34]. Consequently, the strength of evidence underpinning different signs varies considerably.

In addition, there remains a lack of quantitative scoring. While signs are stratified by specificity where known, the tool does not yet assign likelihood ratios or diagnostic scores. This is because the existing literature is inconsistent in providing likelihoods of specified signs that would match across studies.

Cultural and contextual factors may also play a role in FND diagnosis, which was not explored due to the lack of evidence. Some signs, especially in FCD (e.g., bringing written notes), may vary in prevalence or interpretation across health systems and cultures.

Finally, the tool has not yet been formally validated in clinical trials or diagnostic audits, though it is structured to facilitate such future work.

## 8. Conclusions

This study synthesizes the existing clinical literature on FND into a practical, accessible Clinical Decision Tool that emphasizes positive diagnosis across subtypes. By organizing reproducible signs into a structured reference, it empowers clinicians to confidently identify FND using observable features rather than diagnostic exclusion.

In doing so, this tool offers an opportunity to enhance diagnostic accuracy and speed, minimize unnecessary investigations, improve patient understanding and treatment engagement, and lay the foundation for standardized training and future digital decision support tools.

Future directions include the tool’s validation in real-world settings, integration into electronic health systems, and development into interactive digital formats for frontline clinicians.

## Figures and Tables

**Table 1:** Positive clinical signs identified across FND subtypes.

FND Type	Positive Sign
Functional Cognitive Disorder	Absence of the Head Turning to Companion
	Attending Memory Clinic Unaccompanied
	Better Delayed Than Immediate Recall
	Bringing a List of Complaints
	Metacognitive Inconsistency
	Attending Memory Clinic Unaccompanied
	Better Delayed Than Immediate Recall
Functional Gait Disorder	Astasia–Abasia
	Bizarre or Exaggerated Gait Patterns
	Chair Test
	Dragging or Buckling Without True Weakness
	Huffing and Puffing Sign
	Improved Performance with Distraction
	Incongruent Task Response
	Inconsistency Across Contexts
	Preserved Reflexes Despite Instability
	Sudden Onset Without Injury
Functional Limb Weakness	Abductor Sign
	Collapsing/Give-Way Weakness
	Entrainable Weakness
	Hoover’s Sign
	Improved Function in Emergencies
	Normal Movement Under Distraction
Functional Myoclonus	Absence of Reflex Correlates
	Inconsistent Bereitschaftspotential
	Sudden Onset and Fluctuation
	Triggered by Attention or Emotion
	Variability in Pattern
Functional Parkinsonism	Absent Cogwheeling
	Distractibility of Tremor or Bradykinesia
	Excessive Effort or Sighing
	Lack of Finger Tremor
	No Decrement on Repetition
	Normal Arm Swing
	Speech Abnormalities
	Sudden, Maximal Onset
	Tremor Across Postures
Functional Tremor	Co-activation Sign
	Distractibility
	Enhanced by Attention
	Entrainment
	Lack of Finger Tremor
	Tremor Inhibition
	Variable Frequency and Axis
Nonepileptic Seizures	Asynchronous Limb Movements
	Closed Eyes During Episode
	Lack of Postictal Confusion
	Long Duration (>2 min)
	No Injury or Tongue Bite
	Pelvic Thrusting/Rolling
	Resistance to Eye Opening
	Side-to-Side Head Movements
	Variable Responsiveness

## Data Availability

No new data were created or analyzed in this study. Data sharing is hence not applicable to this article.
